# Instituting a Curriculum for Cardio-Obstetrics Subspecialty Fellowship Training

**DOI:** 10.14797/mdcvj.1101

**Published:** 2022-06-03

**Authors:** Anum S. Minhas, Sarah A. Goldstein, Arthur Jason Vaught, Jennifer Lewey, Cary Ward, Steven P. Schulman, Erin D. Michos

**Affiliations:** 1 Johns Hopkins University School of Medicine, Baltimore, Maryland, US; 2 Duke University School of Medicine, Durham, North Carolina, US; 3 University of Pennsylvania, Philadelphia, Pennsylvania, US; 4 Johns Hopkins Bloomberg School of Public Health, Baltimore, Maryland, US

**Keywords:** cardio-obstetrics, education, curriculum, training

## Abstract

Maternal mortality is rising in the United States, and cardiovascular disease is the leading cause. Adverse pregnancy outcomes such as preeclampsia and gestational diabetes heighten the risk of cardiovascular complications during pregnancy and the peripartum period and are associated with long-term cardiovascular risks. The field of cardio-obstetrics is a subspecialty within adult cardiology that focuses on the management of women with or at high risk for heart disease who are considering pregnancy or have become pregnant. There is growing recognition of the need for more specialists with dedicated expertise in cardio-obstetrics to improve the cardiovascular care of this high-risk patient population. Current recommendations for cardiovascular fellowship training programs accredited by the Accreditation Council for Graduate Medical Education involve establishing core competency in the knowledge of managing heart disease in pregnancy. However, little granular detail is available of what such training should entail, which can lead to knowledge gaps. Additionally, dedicated advanced subspecialty training in this area is not commonly offered. Multidisciplinary collaborative teams have been shown to improve outcomes in cardiac patients during pregnancy, and cardiovascular fellows-in-training interested in cardio-obstetrics should have the opportunity to participate in and contribute to a pregnancy heart team. In this document, we describe a proposed specialized cardio-obstetrics training pathway that could serve to adequately prepare trainees to competently and comprehensively care for women with cardiovascular disease before, during, and after pregnancy.

## Introduction

Over the past several decades, the United States (US) is the only developed country in which maternal mortality has worsened.^1^ During this time, the burden of cardiovascular disease (CVD) affecting maternal mortality has increased while obstetrical causes of death have decreased.^2^ In fact, CVD has become the leading cause of pregnancy-related maternal mortality in the US.^2^ This has occurred against a backdrop of advancing maternal age, rising prevalence of cardiovascular comorbidities, acquired heart disease among women of childbearing age, improved pediatric outcomes for patients with congenital heart disease, and persistent racial and socioeconomic disparities, making it critical for clinicians to expand their knowledge and improve care delivery for maternal cardiovascular health.

Up to two-thirds of pregnancy-related deaths due to CVD are considered preventable.^3^ The majority of these deaths are considered to be caused by provider and health systems factors that lead to missed or delayed diagnoses, delay of effective treatments, and lack of communication between providers. In one study of pregnant women with known CVD, almost half of all serious cardiac events were related to factors such as lack of adequate preconception counseling, inappropriate treatment, delay in treatment, late recognition of cardiac deterioration, and failure to identify a cardiac condition as high risk. Furthermore, this study assessed these events to be potentially preventable with adequate provider education and experience.^4^

Additionally, an increasing burden of adverse pregnancy outcomes (APOs) such as preeclampsia, preterm delivery, gestational diabetes, and intrauterine growth restriction has been associated with elevated risk of future early onset CVD among women.^5,6,7,8^ Therefore, it is important to recognize the potentially modifiable cardiometabolic risk factors associated with APOs prior to conception and attempt to mitigate these risks in patients. Recognizing the increased CVD risk postpartum in patients with APOs is essential in risk stratification and promoting optimal lifelong cardiovascular health metrics.

The field of cardio-obstetrics, a subspecialty within adult cardiology that focuses on the management of women with or at high risk of heart disease who are considering pregnancy or are currently pregnant, is rapidly expanding in response to patient needs.^9^ However, there are no existing curricula for cardio-obstetrics training, and this area is not included in the Core Cardiovascular Training Statement (COCATS) put forth by the American College of Cardiology (ACC), which outlines requirements for training in adult cardiovascular medicine.^9,10^ In this review, we describe a proposed specialized cardio-obstetrics training pathway for fellows-in-training (FITs) that could serve to adequately prepare trainees to competently and comprehensively care for women with CVD before, during, and after pregnancy. The aim of this proposed program is for trainees to complete a dedicated 1-year clinical and research subspecialty training program to achieve expertise in cardio-obstetrics.

## Core Components of Cardio-Obstetrics Training

The interest in cardio-obstetrics has surged in recent years, leading to multiple centers with dedicated cardiologists who have subspecialty expertise in cardio-obstetrics, and this surge is likely to continue.^6^ However, there is little formal training in this novel area of cardiovascular practice. The Accreditation Council for Graduate Medical Education (ACGME), which oversees cardiovascular fellowship programs, includes as one of their core competencies for fellows the prevention, evaluation, and management of heart disease in pregnancy.^11^ However, the ACGME does not provide any further specific details of what such training might entail.

The ACC’s COCATS guidelines provide detailed recommendations on the requirements for training in adult cardiovascular medicine.^9,10^ These guidelines advise fellows to learn the impact of pregnancy in patients with adult congenital heart disease (ACHD) and heart failure. They also recommend exposure to an obstetrical clinic that evaluates pregnant patients with heart disease to understand the interdisciplinary approach to caring for patients during a high-risk pregnancy. The COCATS document falls short in outlining any specific training pathway rotations for fellows who desire to develop advanced expertise in the area of cardio-obstetrics,^9,10^ and to date, dedicated advanced subspecialty training in this area is not widely available.

**Figure 1:**
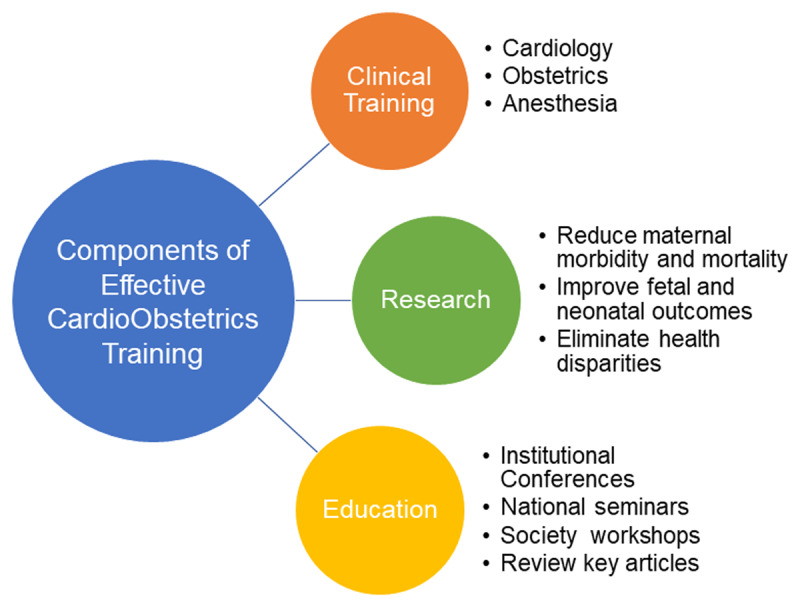
Components of effective cardio-obstetrics training.

Similar to the established COCATS requirements for general cardiology fellowship training and for subspecialty training in interventional cardiology, electrophysiology, and advanced heart failure, there is a need for a dedicated advanced training pathway in cardio-obstetrics.^9,10^ Key components of comprehensive cardio-obstetrics training include clinical training, research, and education (Figure 1).

Formal training in cardio-obstetrics outside of established cardiovascular fellowship programs is also limited, although continuing medical education (CME) programs focused on this topic have recently emerged. More structured education and joint development of core competencies between the ACC, American Heart Association (AHA), and the American College of Obstetrics and Gynecology are necessary to further advance this emerging field.

### Clinical Training

Clinical training in cardio-obstetrics should be based on competency, with specific conditions that the trainee should be exposed to and determined to be competent in managing. Table 1 lists some common general and congenital cardiovascular conditions that should be addressed. Additionally, an understanding of normal physiologic and imaging (ie, echocardiographic) changes during pregnancy, labor, and the peripartum should be reached. Competencies to be achieved in training should include how to (1) perform preconception risk stratification using established tools and risk scores (such as the modified World Health Organization^12^ and CARPREG II^13^ classification schemes); (2) optimize cardiovascular health prior to pregnancy; (3) determine an appropriate antenatal monitoring and delivery plan based on the patient’s cardiovascular complexity in conjunction with the obstetrical team; (4) identify and treat cardiovascular complications that arise during pregnancy and the postpartum period (commonly referred to as the “fourth trimester”); and (5) effectively transition care hand-off back to the primary care physician or obstetrician, if appropriate. Training competencies should also aim to educate trainees on other common adverse outcomes in pregnancy that can affect the cardiovascular system, such as postpartum hemorrhage.

**Table 1:** Conditions expected to be encountered during cardio-obstetrics training.


ADULT CARDIOLOGY CONDITIONS DURING PREGNANCY	

**General cardiology**	Normal cardiovascular physiology of pregnancy

	Hypertensive disorders in pregnancy

	Cardiovascular risks associated with contraception

	Valvular heart disease

	Coronary artery disease (including spontaneous coronary artery dissection)

	Aortopathies

	Pulmonary hypertension

**Heart failure**	Peripartum cardiomyopathy

	Dilated cardiomyopathy

	Restrictive cardiomyopathy

	Hypertrophic cardiomyopathy

	Ischemic cardiomyopathy

**Electrophysiology**	Benign ectopy

	Ventricular arrhythmias

	Supraventricular arrhythmias

	Genetic disorders

**Imaging**	Normal cardiac structural/functional of pregnancy

	Limitations and risks of cardiovascular imaging during pregnancy

ADULT CONGENITAL HEART DISEASE DURING PREGNANCY*	

**Simple complexity**	Small unrepaired secundum atrial septal defect

	Small unrepaired perimembranous ventricular septal defect

	Mild isolated congenital pulmonic stenosis

	Repaired patent ductus arteriosus

	Repaired atrial or ventricular septal defect

**Moderate complexity**	Partial or total anomalous pulmonary venous return

	Anomalous coronary artery

	Atrioventricular septal defect

	Congenital aortic or mitral valve stenosis

	Coarctation of the aorta

	Ebstein anomaly

	Ostium primum atrial septal defect

	Moderate or large unrepaired secundum atrial septal defect, patent ductus arteriosus or ventricular septal defect

	Tetralogy of Fallot

**Severe complexity**	Cyanotic congenital heart defect

	Fontan circulation/single ventricle physiology

	Eisenmenger physiology

	Transposition of the great arteries

	Truncus arteriosus


*Based on American College of Cardiology/American Heart Association anatomic classification system.

**Figure 2:**
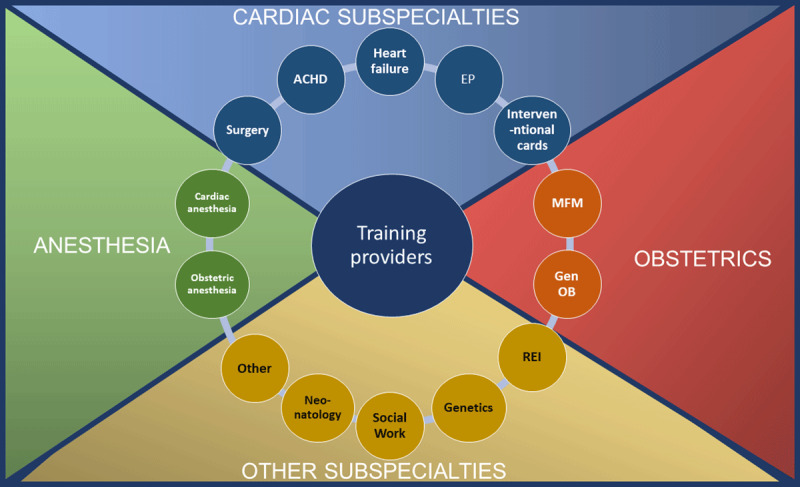
Team-based learning across medical specialties.

Ultimately, optimal care of the cardiac patient during pregnancy requires a team-based approach, including practitioners from cardiology, obstetrics, anesthesia, and other medical subspecialties.^14^ Concurrently, subspecialty cardio-obstetrics training necessitates dedicated exposure to and training with experts in the cardiac subspecialties of ACHD, heart failure, electrophysiology, structural/interventional, and cardiac surgery as well as with experts in the gynecological and obstetrical fields of reproductive endocrinology and infertility, general obstetrics, maternal fetal medicine (MFM), neonatology, and cardiac and obstetrics anesthesia (Figure 2). Other experts, such as pulmonologists, infectious disease doctors, and lipidologists, may also be part of the patient’s care team as clinically indicated. Exposure to the team-based care provided by social workers, geneticists, and pharmacists provides important context to caring for mother and baby. Ultimately, the individual care team for a given patient may involve any additional medical specialties as needed. As in all cardiology training, good communication with nursing is crucial for optimal patient outcomes, and best practices for a team-based approach to care should be modeled during training. Multidisciplinary collaborative teams have been shown to improve outcomes in cardiac patients during pregnancy, and training for FITs in cardio-obstetrics should similarly allow exposure to a pregnancy heart team.^8,13,14,15^

### Research

It is well recognized that CVD is the leading cause of pregnancy-related deaths in the US, with rising maternal morbidity and mortality.^16,17^ Although adverse pregnancy outcomes such as preeclampsia, gestational diabetes, and preterm birth increase long-term CVD risk, much remains to be learned about effective measures for mitigating risk.^5,7,8,18^ Adverse pregnancy outcomes and other forms of maternal morbidity and mortality unfortunately affect underrepresented and historically marginalized women disproportionately.^19,20,21,22^ It is critical, therefore, to advance scientific discovery and research in pregnancy-related heart disease to reduce maternal mortality and morbidity, improve fetal and neonatal outcomes, and reduce health disparities. Trainees seeking to specialize in cardio-obstetrics should gain experience in research focused in this area and appreciate the complexities of care surrounding maternal and fetal health.

Many opportunities exist for research in cardio-obstetrics. Cardiology fellows working in the context of the pregnancy heart team can participate in quality improvement initiatives to improve maternal outcomes, establish care delivery models, and educate providers across the health system. Clinical research opportunities include initiating and maintaining local patient registries and assessing outcomes as well as involvement in national cohort studies and registries that are in development for pregnant patients with CVD. Some of these include the Nulliparous Pregnancy Outcomes (nuMoM2b-Heart Healthy Study), the Heart Outcomes in Pregnancy Expectations (HOPE) Registry, and the Registry of Pregnancy and Cardiac Disease (ROPAC).^23,24,25,26^

Interventions focused on women with APOs to reduce future cardiovascular risk are also an active area of investigation. Epidemiologic and population health studies using large databases—such as the National Inpatient Sample,^19,22,27^ administrative claims databases, and cohort studies such as the HOPE registry,^24^—facilitate understanding. Basic science, translational research, and personalized medicine offer the opportunity to elucidate disease mechanisms and the impact of pregnancy-related CVD on long-term health. Conditions such as peripartum cardiomyopathy may in part be caused by a genetic predisposition, which may impact a woman’s future cardiovascular health as well as the health of her family members.^26^ If research mentors in this field are not available at one’s own training institution, engagement in professional societies can help identify and connect fellows to mentors nationally and internationally.

### Education

While some curricula have been proposed for incorporating women’s cardiovascular health into general cardiology fellowship training, there are no established curricula and didactic learning plans for training specifically in cardio-obstetrics as an advanced subspecialty.^28^ Education in this area should include a mixture of institutional conferences across several disciplines, including cardiology and obstetrics, and attendance at national/international seminars dedicated to cardio-obstetrics. Society workshops and sessions such as those offered by the Society for Maternal Fetal Medicine, the ACC, and the AHA can be potential avenues for education. Lastly, staying abreast with developing literature in the field is indispensable for ongoing lifelong learning in this exponentially growing area of patient care. As mentioned, several dedicated cardio-obstetric CME programs have been created and many offer discounted registration to trainees.

## Clinical Rotations

**Figure 3:**
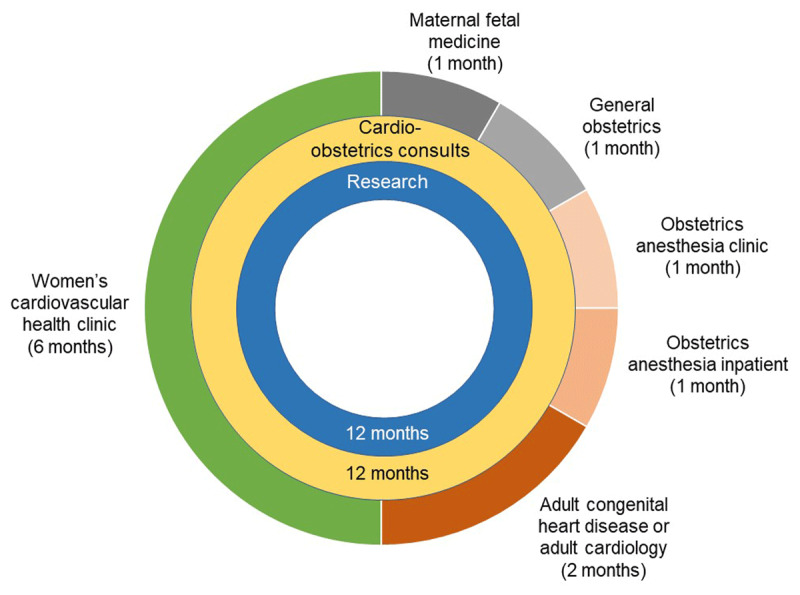
Suggested curriculum for cardio-obstetrics training through general adult and adult congenital heart disease pathways.

For FITs planning to pursue advanced training in this field, we recommend a proposed cardio-obstetrics training pathway that involves 12 months of clinical experiences and focused research and includes both year-long longitudinal activities as well as shorter clinical rotations (Figure 3). All trainees participating in cardio-obstetrics training should complete a year of cardio-obstetrics outpatient clinic and inpatient consultation service, which would typically expose the trainee to the majority of conditions listed in Table 1. They also should participate in a multidisciplinary care management and planning conference (Figure 3).

Other important clinical rotations include time spent in an MFM clinic, on the obstetrics inpatient ward (ie, labor and delivery), and with the obstetrics anesthesia team both in clinic and in an inpatient setting. Even trainees who are not planning to pursue an advanced fellowship in ACHD should spend time in a clinic that cares for this population to gain at least a minimal understanding about managing simple congenital defects. All trainees should also participate in a women’s cardiovascular health clinic that focuses on the long-term cardiovascular effects of APOs.

Additionally, longitudinal research projects focused on relevant topics within the field of cardio-obstetrics should be pursued throughout the year. Research, education, or advocacy efforts may overlap with clinical rotations.

## Forming a Mentorship Team

Mentorship is critical for personal growth and success in academic medicine. Strong mentorship enhances academic development, encourages interest in scholarly work, enables clinical growth, and helps reduce burnout.^29^ Developing as a subspecialist in cardio-obstetrics requires a multidisciplinary mentorship team. At a minimum, a senior clinical cardiologist comfortable providing cardiovascular care to the pregnant woman as well as an MFM specialist are essential for teaching core clinical concepts. For trainees from a general cardiology background, additional clinical mentorship is often necessary from an ACHD-trained expert. For trainees interested in pursuing cardio-obstetric research, additional mentorship from a women’s health researcher is also required. Ultimately, it rests on the trainee to form an ideal mentorship team for clinical, research, and career guidance across specialties. For more on mentorship, see “Starting a Research Career in Cardiology: Advice for Fellows in Training and Early Career Cardiologists” in this issue.

## Funding

Some cardiology fellows may be able to complete cardio-obstetrics training during their 3-year ACGME-accredited cardiology fellowship training by utilizing elective rotations and aligning their outpatient clinic time to have a women’s health focus. However, pursuing an additional dedicated year of subspecialty training outside of cardiology fellowship presents some funding challenges, and opportunities likely vary substantially across institutions.

Typically, sponsoring institutions only have established funding for fellows in ACGME-accredited fellowship programs, which comes from a variety of sources such as federal, state, and private funds. These funding sources often do not include the non-ACGME specialties such as cardio-oncology, sports cardiology, advanced imaging, or cardio-obstetrics, which results in funding for these non-traditional fellowships having to be provided through other intramural or extramural mechanisms. If significant dedicated research time is planned in conjunction with the cardio-obstetric training, potential mechanisms can include the National Institutes of Health T32 training grants or society funding such as the AHA postdoctoral fellowship or the ACC Merck Research Fellowship. Most cardio-obstetrics clinical training programs are likely to be paired with research opportunities, making research funding mechanisms particularly attractive.

If the cardio-obstetrics training is entirely clinically based or conducted at an institution where such research training grants are not available, then other funding sources need to be obtained. Institutional funding is sometimes available through philanthropic support or through offering an instructor faculty position, in which fellows who have completed their general cardiology training can partially support their salaries through independent management of general cardiology patients while training in a non-ACGME-accredited advanced subspecialty program. Unfortunately, given that cardio-obstetrics fellowship training is not ACGME-accredited, funding is not guaranteed and thus remains the responsibility of the institution and individual fellow to secure. As cardio-obstetrics increasingly becomes recognized as its own subspecialty, we hope this pathway will become formalized and accredited by the ACGME, which would help ease funding concerns.

## Career Opportunities

Dedicated training in cardio-obstetrics should provide the skills necessary for a host of career opportunities. Naturally, this includes developing and leading cardio-obstetrics programs or pregnancy heart teams. Such teams have been shown to reduce adverse outcomes and can be used to estimate risk before conception and optimize care during pregnancy and delivery.^14^ Dedicated fellowship training across disciplines allows for smooth integration and successful leadership of a multidisciplinary team.

Additionally, training across subspecialties enhances appreciation of disease pathophysiology and management, essential for collaborative research. Given the robust growth of scientific investigation in cardio-obstetrics and need for in-depth understanding of cardiac disease in pregnancy, dedicated subspecialty training offers unique insights that would be advantageous for a research career.^15^

Given the disparities in maternal health, there is a need for greater advocacy at institutional, state, national, and international levels to improve cardiovascular care of women during and after pregnancy.^18,19,21,22,30^ This includes forming and participating in institutional and state/federal maternal mortality and morbidity review committees and engagement in national and international professional and foundational societies. Formal training in cardio-obstetrics provides education across disciplines and a fresh perspective on the heterogeneous and complex care required for optimal outcomes.

## Developing a Training Program in the Absence of an Existing Cardio-Obstetrics Program

Given that cardio-obstetrics is a newly developing field, it is quite possible that there may be interested trainees at institutions with no existing cardio-obstetrics programs. While this can present a challenge for a trainee, it may also offer a unique opportunity to initiate and develop a new program. We recommend that the trainee first identify one or two faculty members who would support this initiative; often, this will be either the fellowship program director or a leader in women’s cardiovascular health. With assistance from a faculty member, the trainee should then contact leadership in the obstetrics and gynecology division, specifically in MFM, and garner interest in establishing combined pathways for patient care (this could be in the form of a monthly multidisciplinary clinic, clinical conference, or otherwise). Ultimately, forming a relationship with MFM and other relevant medical specialties will be key in developing a cardio-obstetrics program, which can then serve as an opportunity for active clinical learning and research collaborations. While it can be daunting for a trainee to initiate such a program, it can be extremely rewarding for personal growth and development as a leader and gaining expertise in a niche area of cardiovascular medicine.

## Conclusion

Given the expected rise in cardiovascular diseases during pregnancy, the workforce of the future will demand dedicated specialists in managing CVD in this unique population. Therefore, the time has come to increase the amount of training and knowledge in the cardio-obstetrics field. We must foster the number and growth of healthcare practitioners who are comfortable managing women with or at risk for heart disease who are pregnant or planning pregnancy, and who also understand the long-term cardiovascular complications associated with APOs. Healthcare professionals in the field of cardio-obstetrics need to be cognizant of maternal and fetal outcomes and work closely in multidisciplinary heart teams for optimal care.

Currently, there is no formal advanced training in cardio-obstetrics, and the area is often poorly covered in standard cardiology fellowship training. We have therefore outlined a potential blueprint for dedicated cardio-obstetrics subspecialty training for fellows from both general adult cardiology and ACHD backgrounds. Formal accreditation bodies should discuss whether a more formalized cardio-obstetric track, with a certification process, should be created and standardized across training programs. Establishing a structured curriculum for training in cardio-obstetrics is essential for training the next generation of cardiovascular physicians.

## Key Points

- There is a growing need for healthcare professionals with expertise in managing and preventing cardiovascular disease in the setting of pregnancy, but there is currently no dedicated training available.
- The proposed curriculum for fellowship training will provide cardiology fellows competent and comprehensive training in cardio-obstetrics conducted within an integrated team-based approach.
- In addition to achieving clinical competency, training in cardio-obstetrics should incorporate multidisciplinary mentorship and an emphasis on education and research.

## References

[1] GBD 2015 Maternal Mortality Collaborators. Global, regional, and national levels of maternal mortality, 1990-2015: a systematic analysis for the Global Burden of Disease Study 2015. Lancet. 2016 Oct 8;388(10053):1775-1812. doi: 10.1016/S0140-6736(16)31470-2 27733286 PMC5224694

[2] Creanga AA, Syverson C, Seed K, Callaghan WM. Pregnancy-Related Mortality in the United States, 2011-2013. Obstet Gynecol. 2017 Aug;130(2):366-373. doi: 10.1097/AOG.0000000000002114 28697109 PMC5744583

[3] CDC.gov [Internet]. Washington, D.C.: US Department of Health & Human Services; 2022. Pregnancy-Related Deaths in the United States; 2022 Feb [cited 2022 April 23]. Available from: https://www.cdc.gov/hearher/pregnancy-related-deaths/index.html#:~:text=Almost%20two%20thirds%20of%20pregnancy,quality%20care%20can%20save%20lives

[4] Pfaller B, Sathananthan G, Grewal J, et al. Preventing Complications in Pregnant Women With Cardiac Disease. J Am Coll Cardiol. 2020 Mar 31;75(12):1443-1452. doi: 10.1016/j.jacc.2020.01.039 32216913

[5] Hauspurg A, Ying W, Hubel CA, Michos ED, Ouyang P. Adverse pregnancy outcomes and future maternal cardiovascular disease. Clin Cardiol. 2018 Feb;41(2):239-246. doi: 10.1002/clc.22887 29446836 PMC6490154

[6] Ramlakhan KP, Johnson MR, Roos-Hesselink JW. Pregnancy and cardiovascular disease. Nat Rev Cardiol. 2020 Nov;17(11):718-731. doi: 10.1038/s41569-020-0390-z 32518358

[7] Jowell AR, Sarma AA, Gulati M, et al. Interventions to Mitigate Risk of Cardiovascular Disease After Adverse Pregnancy Outcomes: A Review. JAMA Cardiol. 2022 Mar 1;7(3):346-355. doi: 10.1001/jamacardio.2021.4391 34705020 PMC8916981

[8] Parikh NI, Gonzalez JM, Anderson CAM, et al. Adverse Pregnancy Outcomes and Cardiovascular Disease Risk: Unique Opportunities for Cardiovascular Disease Prevention in Women: A Scientific Statement From the American Heart Association. Circulation. 2021 May 4;143(18):e902-e916. doi: 10.1161/CIR.0000000000000961 33779213

[9] Halperin JL, Williams ES, Fuster V. COCATS 4 Introduction. J Am Coll Cardiol. 2015 May 5;65(17):1724-33. doi: 10.1016/j.jacc.2015.03.020 25777643

[10] Halperin JL, Williams ES, Fuster V, et al. ACC 2015 Core Cardiovascular Training Statement (COCATS 4) (Revision of COCATS 3): A Report of the ACC Competency Management Committee. J Am Coll Cardiol. 2015 May;65(17):1721-1723. doi: 10.1016/j.jacc.2015.03.017

[11] ACGME.org [Internet]. Chicago, IL: Accreditation Council for Graduate Medical Education; c2022. ACGME Program Requirements for Graduate Medical Education in Cardiovascular Disease; 2020 Jul 1 [cited 2022 Apr 15]. Available from: https://www.acgme.org/globalassets/PFAssets/ProgramRequirements/141_CardiovascularDisease_2020.pdf

[12] Regitz-Zagrosek V, Roos-Hesselink JW, Bauersachs J, et al. 2018 ESC Guidelines for the management of cardiovascular diseases during pregnancy. Eur Heart J. 018 Sep 7;39(34):3165-3241. doi: 10.1093/eurheartj/ehy340 30165544

[13] Silversides CK, Grewal J, Mason J, et al. Pregnancy Outcomes in Women With Heart Disease: The CARPREG II Study. J Am Coll Cardiol. 2018 May 29;71(21):2419-2430. doi: 10.1016/j.jacc.2018.02.076 29793631

[14] Davis MB, Arendt K, Bello NA, et al. Team-Based Care of Women With Cardiovascular Disease From Pre-Conception Through Pregnancy and Postpartum: JACC Focus Seminar 1/5. J Am Coll Cardiol. 2021 Apr 13;77(14):1763-1777. doi: 10.1016/j.jacc.2021.02.033 33832604 PMC8238394

[15] Davis MB, Walsh MN. Cardio-Obstetrics. Circ Cardiovasc Qual Outcomes. 2019 Feb;12(2):e005417. doi: 10.1161/CIRCOUTCOMES.118.005417 30773028

[16] CDC.gov [Internet]. Washington, D.C.: US Department of Health & Human Services; 2022. Severe Maternal Morbidity in the United States; 2022 Feb [cited 2022 Apr 15]. Available from: www.cdc.gov/reproductivehealth/maternalinfanthealth/severematernalmorbidity.html

[17] CDC.gov [Internet]. Washington, D.C.: US Department of Health & Human Services; 2022. Pregnancy Mortality Surveillance System; 2017 Nov [cited 2022 Apr 15]. Available from: www.cdc.gov/reproductivehealth/maternalinfanthealth/pmss.html

[18] Minhas AS, Ying W, Ogunwole SM, et al. The Association of Adverse Pregnancy Outcomes and Cardiovascular Disease: Current Knowledge and Future Directions. Curr Treat Options Cardiovasc Med. 2020 Dec;22(12):61. doi: 10.1007/s11936-020-00862-6 35296064 PMC8923621

[19] Minhas AS, Ogunwole SM, Vaught AJ, et al. Racial Disparities in Cardiovascular Complications With Pregnancy-Induced Hypertension in the United States. Hypertension. 2021 Aug;78(2):480-488. doi: 10.1161/HYPERTENSIONAHA.121.17104 34098730 PMC8266726

[20] Johnson JD, Louis JM. Does race or ethnicity play a role in the origin, pathophysiology, and outcomes of preeclampsia? An expert review of the literature. Am J Obstet Gynecol. 2022 Feb;226(2S):S876-S885. doi: 10.1016/j.ajog.2020.07.038 32717255

[21] Fingar KR, Hambrick MM, Heslin KC, Moore JE. Trends and Disparities in Delivery Hospitalizations Involving Severe Maternal Morbidity, 2006–2015: Statistical Brief #243. Healthcare Cost and Utilization Project (HCUP) Statistical Briefs [Internet]. Rockville (MD): Agency for Healthcare Research and Quality (US); 2006 Feb. 2018 Sep 4. PMID: 303719957 30371995

[22] Ijaz SH, Jamal S, Minhas AMK, et al. Trends in Characteristics and Outcomes of Peripartum Cardiomyopathy Hospitalizations in the United States Between 2004 and 2018. Am J Cardiol. 2022 Apr 1;168:142-150. doi: 10.1016/j.amjcard.2021.12.034 35074213 PMC9944609

[23] Haas DM, Ehrenthal DB, Koch MA, et al. Pregnancy as a Window to Future Cardiovascular Health: Design and Implementation of the nuMoM2b Heart Health Study. Am J Epidemiol. 2016 Mar 15;183(6):519-30. doi: 10.1093/aje/kwv309 26825925 PMC4782765

[24] Grodzinsky A, Florio K, Spertus JA, et al. Maternal Mortality in the United States and the HOPE Registry. Curr Treat Options Cardiovasc Med. 2019 Jul 25;21(9):42. doi: 10.1007/s11936-019-0745-0 31342274

[25] Roos-Hesselink J, Baris L, Johnson M, et al. Pregnancy outcomes in women with cardiovascular disease: evolving trends over 10 years in the ESC Registry Of Pregnancy And Cardiac disease (ROPAC). Eur Heart J. 2019 Dec 14;40(47):3848-3855. doi: 10.1093/eurheartj/ehz136 30907409

[26] Goli R, Li J, Brandimarto J, et al. Genetic and Phenotypic Landscape of Peripartum Cardiomyopathy. Circulation. 2021 May 11;143(19):1852-1862. doi: 10.1161/CIRCULATIONAHA.120.052395 33874732 PMC8113098

[27] Minhas AS, Rahman F, Gavin N, et al. Cardiovascular and Obstetric Delivery Complications in Pregnant Women With Valvular Heart Disease. Am J Cardiol. 2021 Nov 1;158:90-97. doi: 10.1016/j.amjcard.2021.07.038 34452683 PMC8765669

[28] Reza N, Adusumalli S, Saybolt MD, et al. Implementing a Women’s Cardiovascular Health Training Program in a Cardiovascular Disease Fellowship: The MUCHACHA Curriculum. JACC Case Rep. 2020 Jan 15;2(1):164-167. doi: 10.1016/j.jaccas.2019.11.033 34316988 PMC8301516

[29] Choi AMK, Moon JE, Steinecke A, Prescott JE. Developing a Culture of Mentorship to Strengthen Academic Medical Centers. Acad Med. 2019 May;94(5):630-633. doi: 10.1097/ACM.0000000000002498 31026234 PMC6493700

[30] Shah NS, Kershaw KN, Khan SS. The Intersection of Nativity With Race and Ethnicity in Preeclampsia-Broadening the Assessment of Social Determinants of Maternal Health. JAMA Netw Open. 2021 Dec 1;4(12):e2140674. doi: 10.1001/jamanetworkopen.2021.40674 34928363

